# Leveraging the Global Health Service Partnership Model for Workforce Development in Global Radiation Oncology

**DOI:** 10.1200/JGO.2017.010066

**Published:** 2017-12-15

**Authors:** Omoruyi Credit Irabor, Vanessa Bradford Kerry, Juliet Matton, Wilfred Ngwa

**Affiliations:** **Omoruyi Credit Irabor,** Dana Farber Cancer Institute; **Wilfred Ngwa**, Dana Farber Cancer Institute and Brigham and Women’s Hospital; **Omoruyi Credit Irabor**, **Vanessa Bradford Kerry**, and **Wilfred Ngwa**, Harvard Medical School; **Vanessa Bradford Kerry**, Massachusetts General Hospital, Boston; and **Juliet Matton** and **Wilfred Ngwa**, University of Massachusetts, Lowell. MA.

## Abstract

A major contributor to the disparity in cancer outcome across the globe is the limited health care access in low- and middle-income countries that results from the shortfall in human resources for health (HRH), fomented by the limited training and leadership capacity of low-resource countries. In 2012, Seed Global Health teamed up with the Peace Corps to create the Global Health Service Partnership, an initiative that has introduced a novel model for tackling the HRH crises in developing regions of the world. The Global Health Service Partnership has made global health impacts in leveraging partnerships for HRH development, faculty activities and output, scholarship engagement, adding value to the learning environment, health workforce empowerment, and infrastructure development.

## INTRODUCTION

Cancer is on the rise globally, and incidence in the sub-Saharan Africa region is predicted to increase by 85% in 2030.^1^ Overall, patient fatality for all cancers is 75% for low-income countries in Africa, 72% in low- and middle-income countries (LMICs), and 64% in the upper- and middle-income countries, significantly higher than the 46% average in high-income countries (HICs).^2^ A major contributor to this disparity is the unequal access to health care resulting from the shortfall in human resources for health (HRH), fomented by the limited training and leadership capacity of low-resource countries.^2^ A need-based analysis in 2013 estimated the world to be short of 17.4 million health workers (including 2.6 million doctors and > 9 million nurses) and forecasted a shortage of 18 million by 2030,^3^ affecting both HICs and LMICs.^3,4^ This scarcity has affected health systems across the globe in varying degrees. Although the absolute deficits are significant in Southeast Asia because of its population size, the disparity between the global burden of disease and available health care professional workforce is extremely stark in Africa.^5^ There, the continent has 25% of the global burden of disease but only 3% of the world’s health care workforce. Africa’s HRH deficit has led to the emergence of a few not-for-profit organizations with the aim to support workforce development within the region. One such initiative is Seed Global Health,^6^ started in 2011 with a focus to provide nursing and medical training support in resource-constrained countries. Seed Global Health teamed up with the Peace Corps to create the Global Health Service Partnership (GHSP),^7^ an initiative that has introduced a novel model for tackling the HRH crises in developing regions of the world. In 2016, Seed Global Health and other stakeholders shared their model and approach to global health and cancer control at the Global Health Catalyst (GHC) Summit at Harvard Medical School.^8^ On the basis of the proceedings of the conference, this article explores the GHSP model for advancing the development of a global health workforce from an oncology perspective, appraising its impact and potential for closing the global cancer care disparity.

## THE HRH CRISIS IN AFRICA

The 2006 World Health Report indicated 36 of the 57 countries that have a health workforce crisis globally to be in the African region.^4^ Although the continent is home to approximately 11% of the world’s population, it retains only 3% of global health workers, significantly lower than a region like North America, where the United States and Canada host 37% of the global health workforce for only 14% of the world’s population.^5^ In 2011, there were an estimated two physicians for every 10,000 people in sub-Saharan Africa, compared with the global average of 15 per 10,000.^9^ The statistics were even gloomier for the nursing profession in Africa; there are 12 nurses per 10,000 population, compared with the world average of 33 per 10,000.^10^

Brain drain is one of the most significant influences for the human resource crisis in sub-Saharan Africa, made up of both push and pull factors, which have been well delineated.^11,12^ Push factors are inherent problems within a country or health system that impede desirable training, employment, and retention incentives for health professionals. These forces include budgetary constraints for health professional education or running the health sector, poor remuneration, and lack of nonfinancial incentives such as social recognition, workplace safety, technology, and/or political stability, for example.^11,12^ Pull factors are external to a country or health system, usually including better standards of living or quality of life, higher salaries, access to advanced technology, and more political stability in other countries that attract talent from less-resourced areas.^11,12^ The costs of this outflow of health workers can be considerable and also posit an ethical issue.^12^ When less-resourced nations pay to educate their health care workers only to have them leave for developed countries, they are, in effect, subsidizing wealthier nations.^12^ A recent analysis estimates the loss of returns from investment for all doctors from selected sub-Saharan African countries currently working in more resourced nations at $2.17 billion, ranging from $2.16 million for Malawi to $1.41 billion for South Africa.^12^ Correcting these losses requires a delicate balancing act that protects the right of individual workers to legally migrate, while ensuring that global health care needs are met.^12^ However, with little incentive for health professionals to migrate from HICs to LMICs or remain in LMICs, such a balance might not be easy to achieve.

## THE GHSP MODEL

In response to this unmet need, Seed Global Health partnered with the US Peace Corps to launch the GHSP. GHSP is an innovative public-private partnership and global health program that sends faculty to medical and nursing schools in under-resourced settings, with the aim to improve the HRH capacity, strengthen global health systems, and ultimately save lives.^13^ The initiative counters the brain drain by creating incentives for the deployment of health professionals from wealthy countries to poorer ones until there is a net balance in the outflow and inflow of health practitioners. This vision is, in the words of its founders (Kerry, Auld, and Farmer) published before its creation in 2012^14^:

We envision this program as an International Health Service Corps (IHSC), through which health care workers would engage in medical-service and capacity-building partnerships overseas in exchange for health-related graduate school scholarships and forgiveness of student loans. This effort should be targeted to health care providers in the United States and partner countries who are committed to serving the poor.^14^

**Fig 1:**
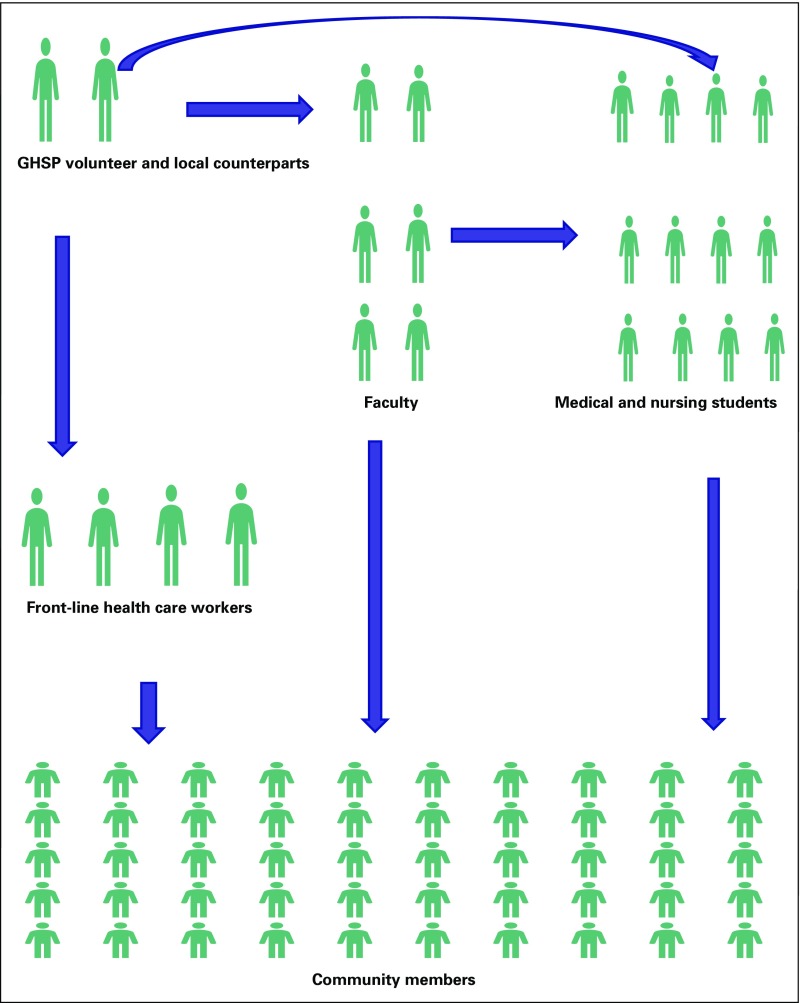
The Global Health Service Partnership (GHSP) train-the-trainer model.

Deployed US health professionals serve as clinical faculty, formally and informally teaching students and house-staff through daily rounds on patients and separate regularly scheduled didactic sessions and courses.^7,13^ The model aims to enhance existing clinical training systems and structures through the development and implementation of innovative teaching tools, clinical guidelines, treatment protocols, and continuing education programs in partnership with host country faculty.^7,13^ GHSP volunteers work in close collaboration with in-country faculty to help ensure they integrate into and foster an efficient and culturally sensitive educational environment (Fig 1). The selection process for GHSP placements is through a consultative process in partnership with the Ministries of Health and of Higher Education, their commitment to strengthening their health care systems, a strong in-country President's Emergency Plan for AIDS Relief presence, and committed local implementing partners.^13,15,16^ A mapping exercise is undertaken with the United States and international partners to reach rapid consensus on priority countries for rollout of the pilot. GHSP’s aim is to create a continuum of health professionals who can teach to the country’s disease burden and can serve as educators in the health and education systems of their countries (Fig 1).

Each cohort of health professional must include board-eligible or board-certified doctors and nurses experienced as educators in core specialties and awarded medical or nursing licenses in the countries where they work, to ensure they are fully qualified and approved. To facilitate health professionals to be able to serve, GHSP helps ensure that educational debt would not preclude being able to serve. The program, through private philanthropy raised by Seed, provides up to $30,000 of debt repayment for each year served. GHSP is also aiming to create specific partnerships with academic institutions to create a structured sabbatical program and to help recruit midcareer health professionals. 

## ASSESSMENT OF THE GHSP

In the past, attempts to counter the brain drain through the deployment of expatriate health workers from developed to low-resource nations were largely abandoned for reasons of cost and sustainability.^5^ Thus, innovative initiatives for HRH development in LMICs must be evaluated for sustainability. Other principal domains for assessment include: the activities and output of faculty, value added to the learning environment, engaged scholarship, and empowerment of health workers, measured against the output and pace needed to close the HRH divide within a specified time frame.

### Sustainability Through Partnership

As opposed to previous projects sustained solely by either governments or charity groups, the GHSP leverages diverse partners for sustainability. Partners include government agencies such as President's Emergency Plan for AIDS Relief and the Centers for Disease Control and Prevention, American health and education institutions, as well as private individuals and organizations, which fund the loan repayment and other critical complementary activities. US private corporations and individuals who incorporate the Global Health Service Corps receive the 501(c)(3) charitable, not-for-profit designation by the Internal Revenue Service. The charitable designation by the Internal Revenue Service, rather than sheer good will, is what incentivizes the private sector participation that financially sustains the GHSP model. The GHSP, through this tax-exempt and charitable designation, succeeded in fostering partnerships, which, if maintained, can keep a stream of technical and financial support for its unscaled budgets. Scale-up budgets will, however, require renegotiation with financing partners or new collaborations. This model for partnership has been sustainable, given that it works to the mutual benefit of partners, as opposed to the regular charitable expectations of global health sponsors.

**Fig 2:**
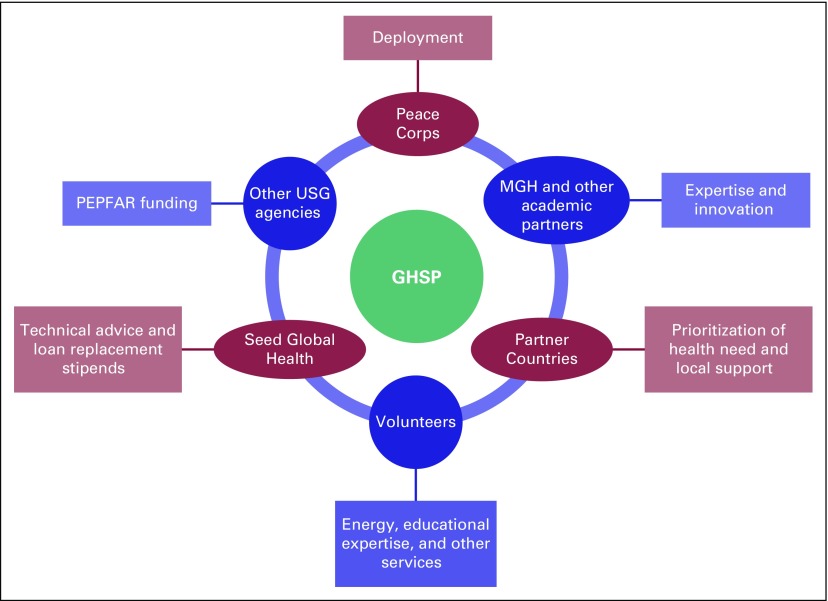
The Global Health Service Partnership (GHSP) partnership model. MGH, Massachusetts General Hospital; PEPFAR, President's Emergency Plan for AIDS Relief; USG, United State Government.

A partnership with US educational institutions to incorporate global health experience in their curriculum allows for a sustained stream of health workers. For example, the Fogarty International Center of the National Institutes of Health has committed to offering extension assignments for Fogarty Fellows wishing to serve as GHSP volunteers, further expanding the pool of potential midcareer participants in GHSP. The Massachusetts General Hospital (MGH) awards Fellowships in Global Clinical Education to volunteers who serve at the GHSP, recognizing the academic educational experience of the volunteer as well as their contribution in service. Figure 2 highlights several partnerships.

### HRH Development

#### GHSP faculty activities and output.

The program was launched in Tanzania, Malawi, and Uganda in July 2013 at 11 institutions, including the University of Malawi College of Medicine, Mzuzu University, and Kamuzu College of Nursing in Malawi; Gulu University, Lira School of Nursing, and Mbarara University of Science and Technology in Uganda; and the Muhimbili University of Health and Allied Sciences, Clinical Officers Training Center, Mirembe School of Nursing, Bugando Medical Center and its affiliate site, Sengerema, in Tanzania. This first cohort consisted of 15 nurses and 16 physicians, selected from a pool of 70 medical and 90 nursing applications.^13^ In the program’s first year, GHSP volunteers provided 32,102 hours of service addressing the training needs and human resource gaps of host countries as determined by the countries and offered 108 courses to 2,853 trainees. The second year, the program grew to deploy 42 volunteers from a larger pool of applicants, provided 53,553 hours of service, and offered 193 courses to 4,366 trainees. Halfway through their third year, GHSP had sent a cumulative total of 105 volunteers and provided a cumulative 104,677 service hours, 344 courses and trainings, and 9,556 trainees (Table 1), with 6 months left in the academic year. Quantifiable expectations are needed to evaluate GHSP output in each host country. However, such expectations have been difficult to set, given the unavailability of recent data on national need and country-level status of human resources for health for Malawi, Uganda, and Tanzania. For example, the limited data on health professional migration from these host countries makes it difficult to analyze the contribution and pace at which the GHSP deployments counteracts the brain drain.

**Table 1:**
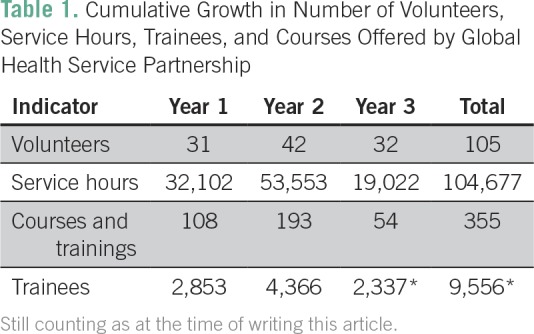
Cumulative Growth in Number of Volunteers, Service Hours, Trainees, and Courses Offered by Global Health Service Partnership

#### Scholarship engagement.

GHSP has contributed to global cancer care through a partnership between Seed Global Health, the Mbarara University of Science and Technology in Uganda, and the Massachusetts General Hospital Cancer Center in the United States, which supports local faculty, residents, and clinical care. Mbarara University of Science and Technology is in southwestern Uganda, a region with few resources, where cancer is a leading cause of death. The university has prioritized creating a cancer center to provide quality diagnosis, treatment, and care for the region. GHSP has responded by sending clinical oncology educators to support scale-up of needed human resources. This contribution also aligns with those of the MGH, who, also through the GHSP platform, has retained clinical faculty in the oncology specialties, using targeted training opportunities for clinicians and other cancer caregivers across the care continuum. MGH and Seed have further strengthened human resource development through the offering of postgraduate scholarships for physicians and nurses, including in subspecialty clinical fellowships of hematology, surgical oncology, and pathology and global nursing fellowships, and have helped provide clinical physician mentorship (in person and remote). Seed and MGH have together invested in pathology training, including scale-up of equipment to support diagnoses. Finally, in Tanzania, GHSP has also supported an ongoing collaborative effort with the University of Dodoma and the Tanzanian Ministry of Health to train health providers and professional students on the simple techniques to prevent cervical cancer.

#### Value added to learning environment and empowerment.

**Fig 3:**
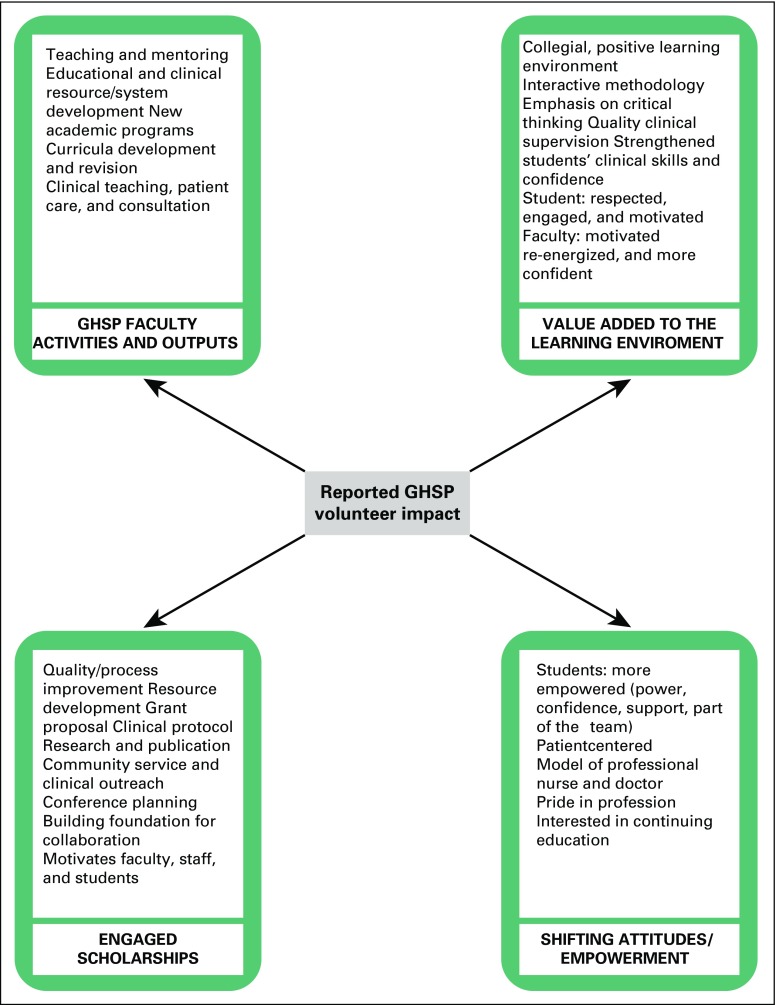
Reported Global Health Service Partnership (GHSP) volunteer impact.

Qualitative data were collected at the end of year 1 through 68 interviews (individual and small group) with 110 stakeholders, including volunteers, institutional leadership, faculty, and students. These data suggest that the GHSP educator added value to the learning environment through enhanced clinical education, quality clinical supervision, and strengthening student clinical skills, critical thinking, and confidence (Fig 3). Values contributed to enhancing self-reported empowerment, confidence, and pride in the profession, as health providers see themselves as competent and capable of delivering health solutions.^13^ This shows that visiting faculty, when vested in a culturally appropriate and locally tailored approach, can contribute to the production of nurses and physicians who are skilled and practice ready when they graduate (Fig 3). There is a need, however, for GHSP to measure the value of care provided by the trained, such as patient outcome and patient satisfaction.

### Infrastructure Development

GHSP has provided support for infrastructure improvement, recognizing that adequate tools are needed for teaching and for health professionals to practice in what they train. Specific to cancer care, GHSP has made contributions to cancer care in partnership with MGH, including supporting core equipment for pathology training. Seed also nurtured a relationship with the American Society for Clinical Pathology and Butaro University in Rwanda to see modest, targeted infrastructure upgrades for cancer diagnosis and management that include microscopes and telepathology in Rwanda. The American Society for Clinical Pathology initiative mainly aims to improve accuracy and clinical relevance of cancer diagnosis in African settings. GHSP, through scholarships for Masters in Medicine,direct mentorships, and training, has concurrently supported human resource capability to ensure laboratory upgrades are well used. These efforts complement again those of MGH, which has helped with the construction of pediatric and adult inpatient wards, infusion chairs, and biosecurity fume hoods for chemotherapy preparation.

## PRELIMINARY FRAMEWORK FOR PARTNERSHIP IN GLOBAL RADIATION ONCOLOGY

An appraisal of these achievements of the GHSP within its first 3 years led to the conceptualization of a GHSP-modeled initiative for global oncology workforce development at the 2016 GHC summit.^8^ Framework for the GHC global oncology workforce development includes the implementation of a volunteer-based program for teleradiation oncology care, research, and education in Africa. The GHC teleoncology platform is open to high-quality direct volunteer support of board-eligible and board-certified oncologists, medical physicists, and other health professionals in US-based institutions for the training of health professionals in the sub-Saharan African region. Expectations are to be proposed and matched with outcomes in the annual GHC summit events.^8^ On the basis of resolutions at the 2016 summit, GHC has gained momentum and launched its first online global oncology certificate course in May 2017, with its first set of volunteers from Harvard Cancer Center and with trainees distributed all over sub-Saharan Africa. Although other online educational activities have existed in the past, the GHC online training is unique for its leverage of partnerships for service output and financial sustenance as well as its focus on oncology practice in specified LMICs. Other anticipated outcomes from the GHC 2016 summit include the establishment and implementation of new cancer centers in Africa (Kenya and Namibia), which are based on incentivized partnership with sponsors and donors, the mobilization of hundreds of diaspora groups for turning brain drain to gain against cancer, and implementing real-time online quality-assurance systems in radiation oncology. Some pioneering partners include the African Organization for Research and Training in Cancer, which will provide a level of coordination and a database of all clinical oncology practitioners in Africa; the Quality Assurance Review Center for imaging and radiotherapy quality assurance; Harvard Cancer Center; and other funding partners. Like the GHSP, its goal is to create a train-the-trainer model in clinical oncology and research to sustain infrastructural investment in cancer management within the sub-Saharan Africa region until the cancer divide is closed.

The GHC also runs the Win-Win initiative, where key stakeholders in the radiotherapy equipment industry, including Varian, Elekta, and TeamBest, have partnered to train and aid entrepreneurs in developing cancer centers in sub-Saharan Africa. Outcomes for the 2016 Win-Win partnership between local African entrepreneurs and the radiotherapy supply end actors will be evaluated in 2017. Multicenter radiation oncology clinical trials initiatives with LMIC cancer centers, through a partnership with the Quality Assurance Review Center, have also been proposed on the GHC platform. With the massive support from radiation oncologists in Nigeria and Tanzania, and the African Organization for Research and Training in Cancer, the GHC is currently priming to foster this collaboration. These partnerships, however, are not intended to be sustained through sheer altruism. In a similar pattern with the GHSP, the GHC global radiation oncology platform looks in the nearest future to formally recognize the academic educational experience of the trainers as well as other volunteers’ contributions in the services of global radiotherapy workforce development. There is, however, the anticipation for the US government and governments of other developed countries to participate in global oncology, even if through a nonfinancial incentivization of global oncology partners such as charitable designations.

## FUTURE PERSPECTIVES

GHSP is currently working to expand to Liberia and Swaziland, with plans to evaluate the impact that its trained trainers have had in their countries’ health and education system and on their future trainees. Meanwhile, the GHC looks forward to expanding the horizons of the Win-Win partnerships to develop and support cancer centers in many more African countries, including Tanzania, Nigeria, Kenya, Cameroon, Rwanda, Ghana, South Africa, Ivory Coast, Namibia, and Jamaica.^8^
